# 21 hypoglycemia cases with hyperinsulinemia

**DOI:** 10.1186/1687-9856-2013-S1-P177

**Published:** 2013-10-03

**Authors:** Yusuke Mizuno, Satsuki Nishigaki, Kengo Miyashita, Akiko Yamamoto, Yasuhiro Naiki, Reiko Horikawa

**Affiliations:** 1 Division of endocrinology and metabolism, National Center For Child Health and Development, Japan

## 

Congenital hyperinsulinemic hypoglycemia (CHI) is the common cause of severe hypoglycemia in infancy. Profound hypoglycemia requires appropriate diagnosis and aggressive treatment to prevent severe and irreversible brain damage. Here we report 21 Japanese hypoglycemia cases with severe hyperinsulinemia.

We report 16 CHI cases, 2 HIHA cases, 1 GSD1b case and 2 PSS cases. 12 cases of CHI had severe episodes of cardiac arrest or seizure in neonatal period, 3 cases of CHI had seizure in infancy, 1 case of CHI had hypoglycemia in infancy. Neonatal-onset CHI cases were treated by octreotide and glucagon at the start. 4 cases of CHI (3 of them had KCNJ11 mutation) were treated as diabetes after pancreatic excision. We will perform pancreatic partial excision to 1 case of CHI who had paternal mutation of ABCC8. 1 case of CHI with ABCC8 mutation is on good control by only diet therapy. We stopped the treatment by diazoxide to 4 cases of CHI until 10 years old. Acuity of the them was varied on the onset. 5 cases of CHI is still treated by diazoxide. HIHA cases had seizure in infancy. They are on good control by diazoxide. GSd1b case had severe hypoglycemia in neonatal period. He was treated as CHI patient by diazoxide. He was diagnosed as GSd1b by hepatomegaly. After live-donor liver transplant, he is on the good control without medicaton. 1 of PSS cases had hypoglycemia in babyhood. He was cured by surgery. 1 of PSS cases had severe hypoglycemia attack in neonatal period. His family did not choose surgery, so he is still treated by diazoxide.

Three-quarter of CHI cases had severe hypoglycemic attack in neonatal period. Some cases needed surgery, some cases could be controlled by internal therapy and could be cured. 5 cases had hyperinsulinemia except CHI cases. Some cases showed hyperammonemia except HIHA. Correct diagnosis for hypoglycemia cases with hyperinsulinemia needs not just blood test but also imaging or genetic testing.

